# A comparative study of the effect of the dose and exposure duration of anabolic androgenic steroids on behavior, cholinergic regulation, and oxidative stress in rats

**DOI:** 10.1371/journal.pone.0177623

**Published:** 2017-06-08

**Authors:** Andressa Bueno, Fabiano B. Carvalho, Jessié M. Gutierres, Cibele Lhamas, Cinthia M. Andrade

**Affiliations:** 1 Programa de Pós Graduação em Medicina Veterinária, Hospital Veterinário, Centro de Ciências Rurais, Universidade Federal de Santa Maria, Santa Maria/RS, Brazil; 2 Programa de Pós Graduação em Ciências Biológicas: Bioquímica Toxicológica, Departamento de Bioquímica e Biologia Molecular, Universidade Federal de Santa Maria, Santa Maria/RS, Brazil; Universidade de Sao Paulo, BRAZIL

## Abstract

The aim of this study was to assess if the dose and exposure duration of the anabolic androgenic steroids (AAS) boldenone (BOL) and stanazolol (ST) affected memory, anxiety, and social interaction, as well as acetylcholinesterase (AChE) activity and oxidative stress in the cerebral cortex (CC) and hippocampus (HC). Male Wistar rats (90 animals) were randomly assigned to three treatment protocols: (I) 5 mg/kg BOL or ST, once a week for 4 weeks; (II) 2.5 mg/kg BOL or ST, once a week for 8 weeks; and (III) 1.25 mg/kg BOL or ST, once a week for 12 weeks. Each treatment protocol included a control group that received an olive oil injection (vehicle control) and AAS were administered intramuscularly (a total volume of 0.2 ml) once a week in all three treatment protocols. In the BOL and ST groups, a higher anxiety level was observed only for Protocol I. BOL and ST significantly affected social interaction in all protocols. Memory deficits and increased AChE activity in the CC and HC were found in the BOL groups treated according to Protocol III only. In addition, BOL and ST significantly increased oxidative stress in both the CC and HC in the groups treated according to Protocol I and III. In conclusion, our findings show that the impact of BOL and ST on memory, anxiety, and social interaction depends on the dose and exposure duration of these AAS.

## Introduction

Anabolic androgenic steroids (AAS) form a large class of synthetic androgens that mimic the effects of male sex hormones such as testosterone and dihydrotestosterone. AAS are widely used by athletes to increase muscle mass and enhance physical performance, and by non-athletes for esthetic reasons. Both young athletes and non-athletes may take 10–100-fold the physiological dose of AAS [1]. Further, several studies have investigated the potentially severe side-effects of AAS abuse [2, 3]. In fact, the number of adolescents using AAS has grown significantly over the past 10 years, with estimates of use ranging between 4–12% among adolescents.

AAS in supraphysiological doses affect several central nervous system- (CNS) related behaviors such as memory, aggression, anxiety, and depression [2, 4]. Studies investigating the mechanisms underlying AAS demonstrated that AAS influence neurotransmission in the CNS by directly affecting the cellular membrane, modulating synthesis and degradation of neurotransmitters, and altering neurotransmitter metabolism [5, 6]. In addition, androgen receptors are expressed in brain structures such as the hippocampus (HC), amygdala, and cerebral cortex (CC). Further, the use of AAS interferes with important signaling and neurotransmission systems, such as glutamatergic [7], cholinergic [8], and opioid systems, that modulate animal behavior [9].

Behavioral responses to AAS depend on several factors, including the chemical structure of the steroid administered, whether a single compound or cocktail is administered, the recipient’s age, and treatment duration [10]. While several controlled studies have described the behavioral changes induced by AAS, these studies primarily used hormone cocktails. Therefore, the aim of this study was to comparatively and separately assess the effects of the AAS boldenone (BOL) and stanazolol (ST) on behavioral tasks to determine if one or both altered learning, memory, anxiolytic-like behavior, and dominant or submissive social behavior. In addition, in an attempt to simulate different user groups, we analyzed the effects of three different treatment protocols that varied in dose and treatment duration to determine their impact on user outcomes.

## Materials and methods

### Animals

Male Wistar rats (45 days of age) weighing +/-200 g were used in the study. The animals were maintained in the Central Animal House of the Federal University of Santa Maria in colony cages at an ambient temperature of 23 ± 2°C and a relative humidity of 45–55% under a 12-h light/dark cycle. The animals had access to a standard rodent pellet diet and water *ad libitum*.

### Experimental treatment protocols

Stanazolol (Estrombol^TM^, Fundación Lab, Argentina) and boldenone undecylenate (Equipoise^TM^, Fort Dodge Lab, USA) treatments were administered according to Protocol I, II and III. Both the vehicle (olive oil) and AAS were administered intramuscularly (a total volume of 0.2 ml) once a week in all three treatment protocols. A total of 90 animals were used to protocol I, II, or III, with rats being randomly allocated to 3 groups: vehicle (VE, n = 10), Boldenone (BOL, n = 10) and stanazolol (ST, n = 10).

**Fig 1:**
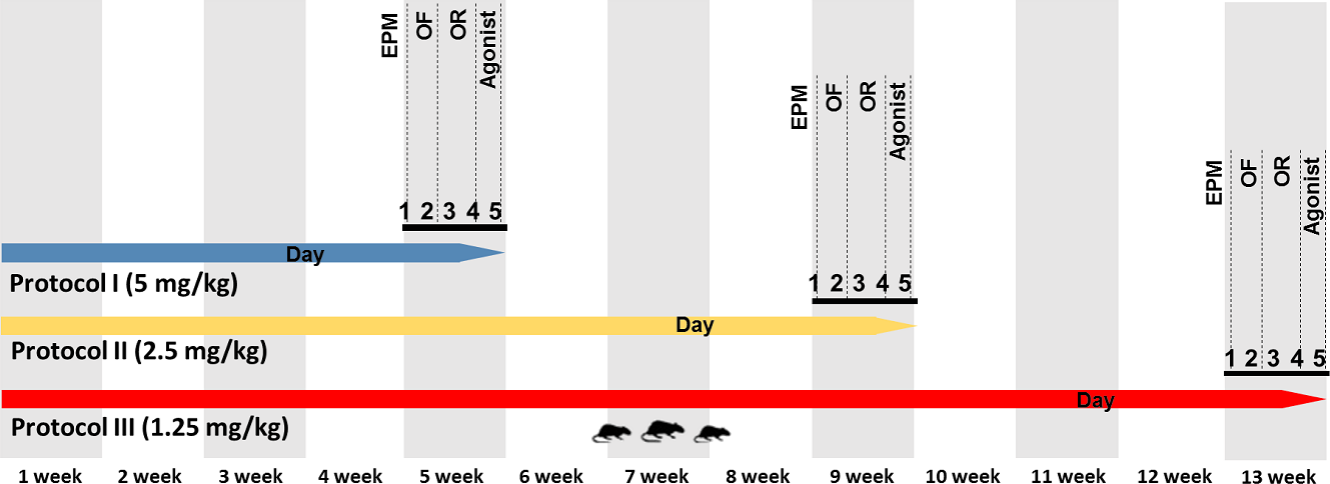
Experimental Protocols: **Protocol I:** Intramuscular injection of vehicle (olive oil, 0.2 ml), stanazolol (ST, 5 mg/kg), or boldenone (BOL, 5 mg/kg) once a week for 4 weeks. **Protocol II:** Intramuscular injection of vehicle (olive oil, 0.2 ml), stanazolol (ST, 2.5 mg/kg), or boldenone (BOL, 2.5 mg/kg) once a week for 8 weeks. **Protocol III:** Intramuscular injection of vehicle (olive oil, 0.2 ml), stanazolol (ST, 1.25 mg/kg) or boldenone (BOL, 1.25 mg/kg) once a week for 12 weeks. Day 1: elevated plus maze task (EPM). Day 2: object recognition task, habituation. Day 3: object recognition task, training. Day 4: object recognition task, testing. Day 5: agonist behavior (resident–intruder paradigm), (n = 10).

Protocol I: 5 mg/kg of ST or BOL for 4 weeks. Protocol II: 2.5 mg/kg of ST or BOL for 8 weeks. Protocol III: 1.25 mg/kg of ST or BOL for 12 weeks. A representative treatment scheme is shown in Fig 1. The animals were anesthetized using halothane before being euthanized by total exsanguination. The CC and HC of each rat were dissected and homogenized in a Tris–HCl 10 mM solution, pH 7.4, on ice [11].

### Behavioral tests

#### Elevated plus maze task

On the first day of the evaluation of the behavioral parameters, anxiolytic-like behavior was evaluated using the elevated plus maze (DAY 1) as previously described [12]. The apparatus consisted of a wooden structure raised to 50 cm from the floor, and was composed of four equally sized arms (two closed arms [walls 40 cm] and two open arms). The rats were placed on the central platform of the maze facing an open arm, and were allowed 5 min to explore the apparatus. The time spent and the number of entries in the open and closed arms were recorded. The apparatus was thoroughly cleaned with 30% ethanol between each session.

#### Open field test

Locomotor and exploratory activities were evaluated in the apparatus used for the object recognition task as previously described [13]. The number of crossings and rearings was assessed on the first day of exposure to the apparatus (habituation day, DAY 2). The animals were transferred to an apparatus with an open field of 100 cm × 100 cm (floor; divided into 16 squares of 6.25 cm^2^ each) × 50 cm (walls). During the 5-min open-field session, the number of crossings and rearings was recorded.

#### Object recognition test

The object recognition task makes use of the spontaneous tendency of a rat to explore its environment and does not require punishment or reward [14]. Here, it consisted of three sessions: habituation (DAY 2), training (DAY 3), and testing (DAY 3), and was conducted as previously described [15]. Briefly, the rats were left to freely explore a square arena (length: 100 cm × 100 cm, height: 50 × 50 cm) for 10 min in the absence of any objects. Twenty-four hours after habituation, the training session was conducted by placing one rat into the arena in which two identical objects (objects A1 and A2) were positioned at two adjacent sides of the arena. The rat was allowed to explore the arena and objects for 10 min. Generally, during the training session, a rat should explore each object for 40–60% of the time spent in the arena, or otherwise be excluded from the experiments. Here, during the test session performed 24 h after the training session, the rats were allowed 10 min to explore the arena in which one of the familiar objects used during the training session was replaced by a novel object (object B). The discrimination index was then calculated, taking into account the difference of time spent exploring the novel (B) and the familiar (A) object x 100 divided by the sum of time spent exploring the novel (B) and the familiar (A), and used as a cognitive parameter ([(Tnovel–Tfamiliar)/(Tnovel X Tfamiliar)]/100) [20]. The objects were made of odorless plastic and were similar in size. Between each trial, the objects and the arena were cleaned with a 30% ethanol solution. The total time spent sniffing or touching each object with the nose and/or forepaws and the number of rearings were analyzed.

#### Agonistic behavior test

The rats were tested for agonistic behavior using the resident–intruder paradigm described by Salas-Ramires et al. [10]. After a 5-min acclimation period, an age- and weight-matched male intruder was placed among the rats in the vehicle, BOL, and ST home cage. The task was recorded and the recordings were analyzed. The duration and number of occurrences of dominant behavior over the intruder (contact team and offensive posturing) and dominant behavior over the territory (number of flank marks) were recorded. Aggressive behavior was determined by the number of attacks and bites over the intruder. Submissive behavior was determined by the frequency of defensive posturing, walking with tail upward, and escape dashes. The intruders were used for more than one behavioral test. All rats were tested during the first 4 h of the dark cycle under dimmed red light conditions to control for circadian influences on behavioral responses [16].

#### Sample preparation for biochemical parameters

The CC and HC were dissected and placed in a 10-mM Tris–HCl solution and 0.1 mM EDTA, pH 7.4, on ice, followed by homogenization in a glass potter in Tris–HCl solution [17]. Aliquots of the homogenate were separated. After centrifugation at 1’500 × *g* at 4°C for 15 min, aliquots of the supernatant were stored at −80°C until use.

#### Measurement of intracellular ROS production

2′-7′-Dichlorofluorescein diacetate (DCFH-DA) levels were determined as markers of intracellular ROS production. Aliquots (50 μl) of the supernatants of the CC and HC were added to a medium containing Tris–HCl buffer (10 mM, pH 7.4) and 1 mM DCFH-DA. After the addition of DCFH-DA, the medium was incubated in the dark for 1 h until fluorescence was measured (excitation at 488 nm and emission at 525 nm, with slit widths of 1.5 nm). DCFH-DA levels were determined using a standard curve consisting of DCF; the results were normalized to the total protein content [18].

#### MDA levels

MDA levels in the CC and HC homogenates were measured using the thiobarbituric acid reactive species (TBARS) method as previously described [19], with minor modifications [20]. Briefly, the reaction mixture contained 200 μl of homogenate or standard (MDA, 0.03 mM), 200 μl of 8.1% sodium dodecylsulfate (SDS), 750 μl of acetic acid solution (2.5 M HCl, pH 3.5), and 750 μl of 0.8% thiobarbituric acid (TBA). The mixtures were heated at 95°C for 90 min. After centrifugation at 1’700 × *g* for 5 min, the absorbance was measured at 532 nm. The MDA tissue levels were expressed as µmol MDA/mg of protein.

#### GSH levels

GSH levels were determined in the CC and HC as previously described [21]. Aliquots of the supernatant (100 μl) adjusted to 1 mg/ml of protein content were added to 85 μl of a phosphate buffer (300 mM, pH 7.4), 50 μl of a 10-mM 5,5′-dithiobisnitrobenzoic acid (DTNB) solution were added, and the reaction was read at 412 nm. The results were expressed as μmol of GSH/mg of protein.

#### NPSH levels

Tissue NPSH levels were determined in the CC and HC as previously described [21]. Briefly, the supernatant was diluted (1:1) with 10% trichloroacetic acid (TCA), homogenized, and centrifuged at 2’000 × *g* for 10 min. Subsequently, the supernatant was incubated with 10 mM DTNB in a final volume of 2 ml, and the absorbance was read at 412 nm. A cysteine solution was used as the reference standard. The NPSH levels were expressed as µmol SH/mg of tissue.

#### Determination of cerebral AChE activity

AChE activity was determined as previously described [22], with a modification of the spectrophotometric method [23]. The reaction mixture (2 ml final volume) contained 100 mM K^+^-phosphate buffer, pH 7.5, and 1 mM DTNB. This method is based on the formation of the yellow anion 5,5′-dithio-bis-acid-nitrobenzoic as measured by its absorbance at 412 nm during a 2-min incubation at 25°C. The enzyme (40–50 μg of protein) was pre-incubated for 2 min. The reaction was initiated by adding 0.8 mM acetylthiocholine iodide (AcSCh). All samples were run in triplicate and enzyme activity was expressed in μmol AcSCh/h/mg of protein. The protein concentration was determined beforehand in a piece of the respective brain tissue: CC, 0.7 mg/ml and HC, 0.8 mg/ml as previously described [24].

#### Protein concentration determination

Protein concentrations were measured by the Coomassie Blue method [25] using bovine serum albumin as the standard.

#### Statistical analysis

The Shapiro-Wilk test was included in the statistical analysis for the determination of the normal distribution of data (SPSS *Statistics 22*.*0*). One-way ANOVA followed by Tukey's test was used for the analysis of behavioral results, stress parameters, and enzyme activity tests. *P<*0.05 was considered significant in all experiments (GraphPad *Prism 5*.*0)*. All data were expressed as the mean ± SEM.

#### Ethical approval

All procedures were performed according to the NIH Guide for the Care and Use of Laboratory Animals and the Brazilian Society for Neuroscience and Behavior (SBNeC) recommendations for animal care. This work was approved by the ethical committee of the Federal University of Santa Maria, Brazil (protocol number 032/2014).

## Results

### Anxiogenic-like behavior of rats treated with BOL and ST according to Protocol I, II, and III

**Fig 2:**
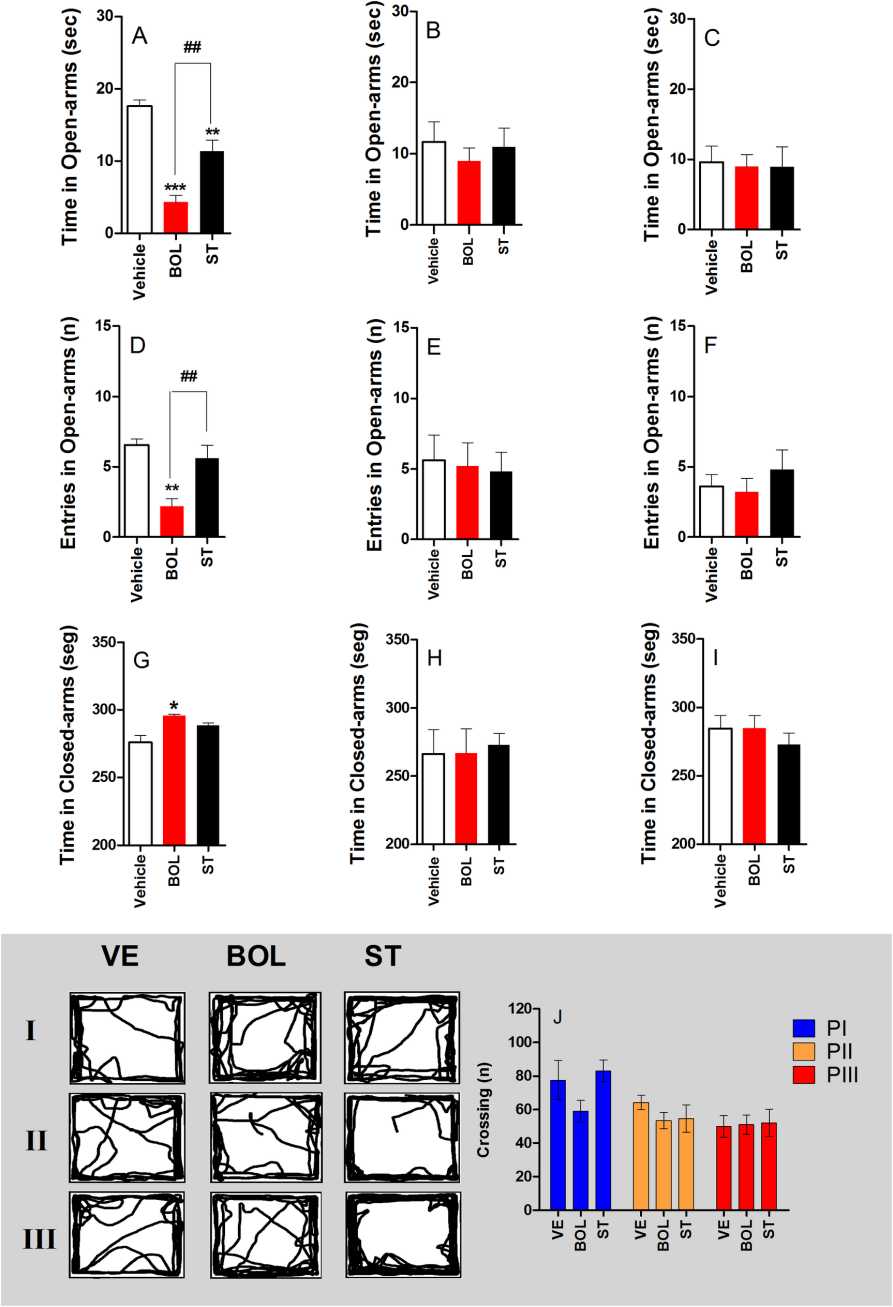
Anxiogenic-like behavior of rats (n = 10) treated intramuscularly with vehicle (olive oil, 0.2 ml), boldenone (BOL), or stanazolol (ST) once a week according to Protocol I (4 weeks, 5 mg/kg), Protocol II (8 weeks, 2.5 mg/kg), or Protocol III (12 weeks, 1.25 mg/kg) as assessed in the elevated plus maze task. Time spent in the open-arms (A, B, and C), entry to open-arms (D, E, and F), time spent in closed-arms (G, H, and I), and crossing numbers (J; P1, P2 and P3). *Denotes significant difference from the vehicle group. # Denotes significant difference between BOL and ST groups.

Fig 2 shows the anxiogenic-like behavior of rats in the elevated plus maze. Rats receiving BOL and ST according to Protocol I showed a decrease in the time spent in the open arms [F_(2,29)_ = 30.53, p<0.001, graph A], while those receiving BOL and ST according to Protocols II and III did not show differences in the time spent in the open-arms [F_(2,29)_ = 0.301, graph B and F_(2,29)_ = 0.027, graph C, respectively]. Rats receiving BOL according to Protocol I showed a decrease in the number of open-arm entries [F_(2,29)_ = 11.14, p<0.01, graph D]. However, rats receiving ST according to Protocol I and those receiving BOL and ST according to Protocol II [F_(2,29)_ = 0.0612, graph E] and III [F_(2,29)_ = 0.517, graph F] did not show a change in the number of open-arm entries. Rats receiving BOL according to Protocol I spent more time in the closed-arm [F_(2,29)_ = 10.86, p<0.01, graph G]. BOL and ST administered according to Protocol II did not affect the time spent in the closed arm [F_(2,29)_ = 0.057, graph H] and III [F_(2,29)_ = 0.557, graph I]. Further, treatment with BOL and ST according to Protocol I, II, or III did not affect crossing numbers [F_(2,29)_ = 1.855, P1; F_(2,29)_ = 0.824, P2 and F_(2,29)_ = 0.025, P3, graph J].

### Dominant behavior over an intruder of rats treated with BOL and ST according to Protocol I, II, and III

**Fig 3:**
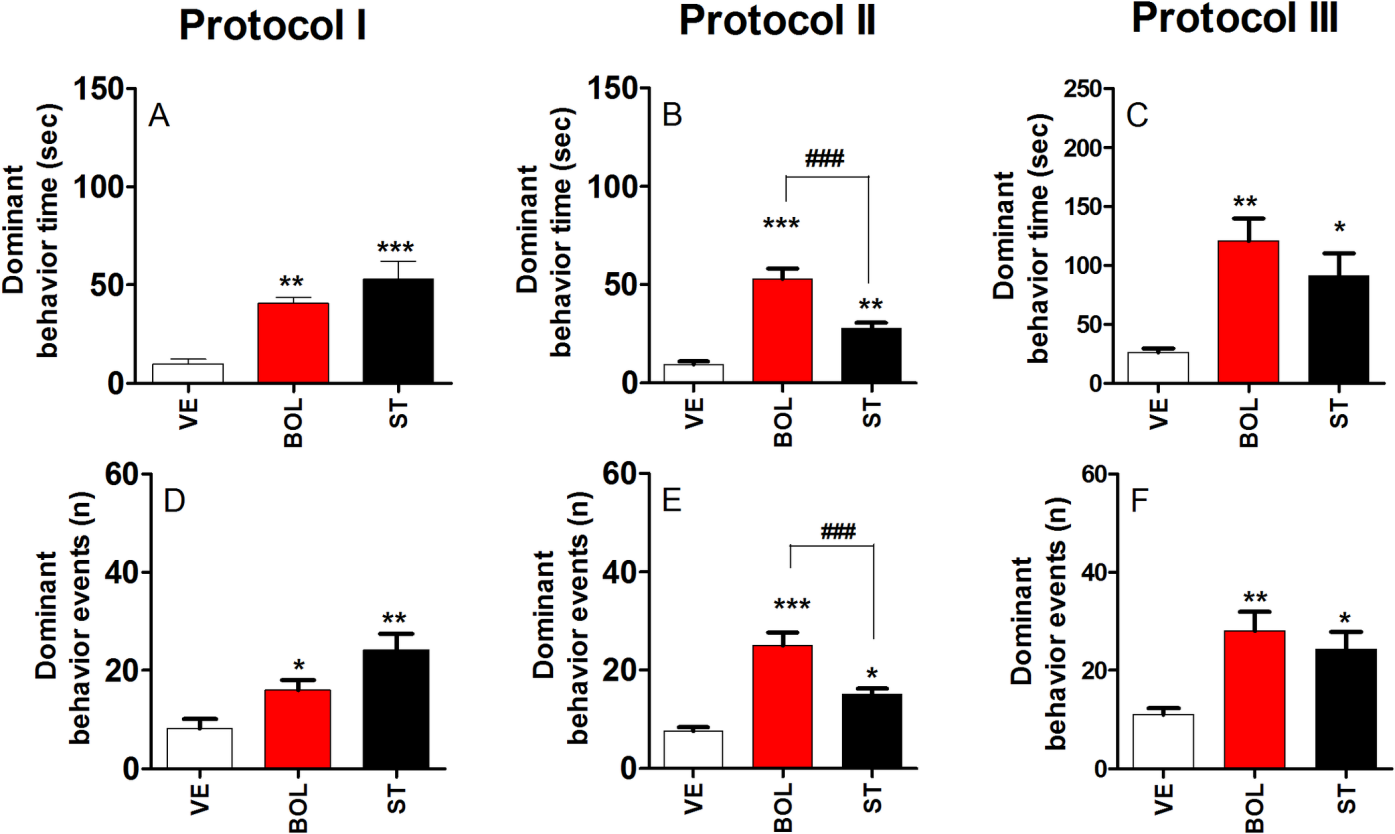
Dominant behavior over the intruder of rats (n = 10) reated intramuscularly with vehicle (olive oil, 0.2 ml), boldenone (BOL), or stanazolol (ST) once a week according to Protocol I (4 weeks, 5 mg/kg), Protocol II (8 weeks, 2.5 mg/kg), or Protocol III (12 weeks, 1.25 mg/kg). Dominant behavior time for Protocol I (A), II (B), and III (C) and dominant behavior events for Protocol I (D), II (E), and III (F). *Denotes significant difference from the vehicle group. # Denotes significant difference between BOL and ST groups.

Fig 3 shows the time (A, B, and C) and events (D, E, and F) of dominant behavior over an intruder after treatment with BOL or ST according to Protocol I, II, and III. The duration of dominant behavior was increased for rats treated with BOL and ST according to Protocol I, II, and III [F_(2,29)_ = 14.74, p<0.001, graph A; F_(2,29)_ = 36.22, p<0.001, graph B; and F_(2,29)_ = 9.623, p<0.01, graph C, respectively]. Similarly, rats treated with BOL and ST according to Protocol I, II, and III, showed an increase in dominant behavior events [F_(2,29)_ = 10.04, p<0.01, graph D; F_(2,29)_ = 25.49, p<0.001, graph E; F_(2,29)_ = 8.263, p<0.01, graph F, respectively].

### Territorial dominant behavior of rats treated with BOL and ST according to Protocol I, II, and III

**Fig 4:**
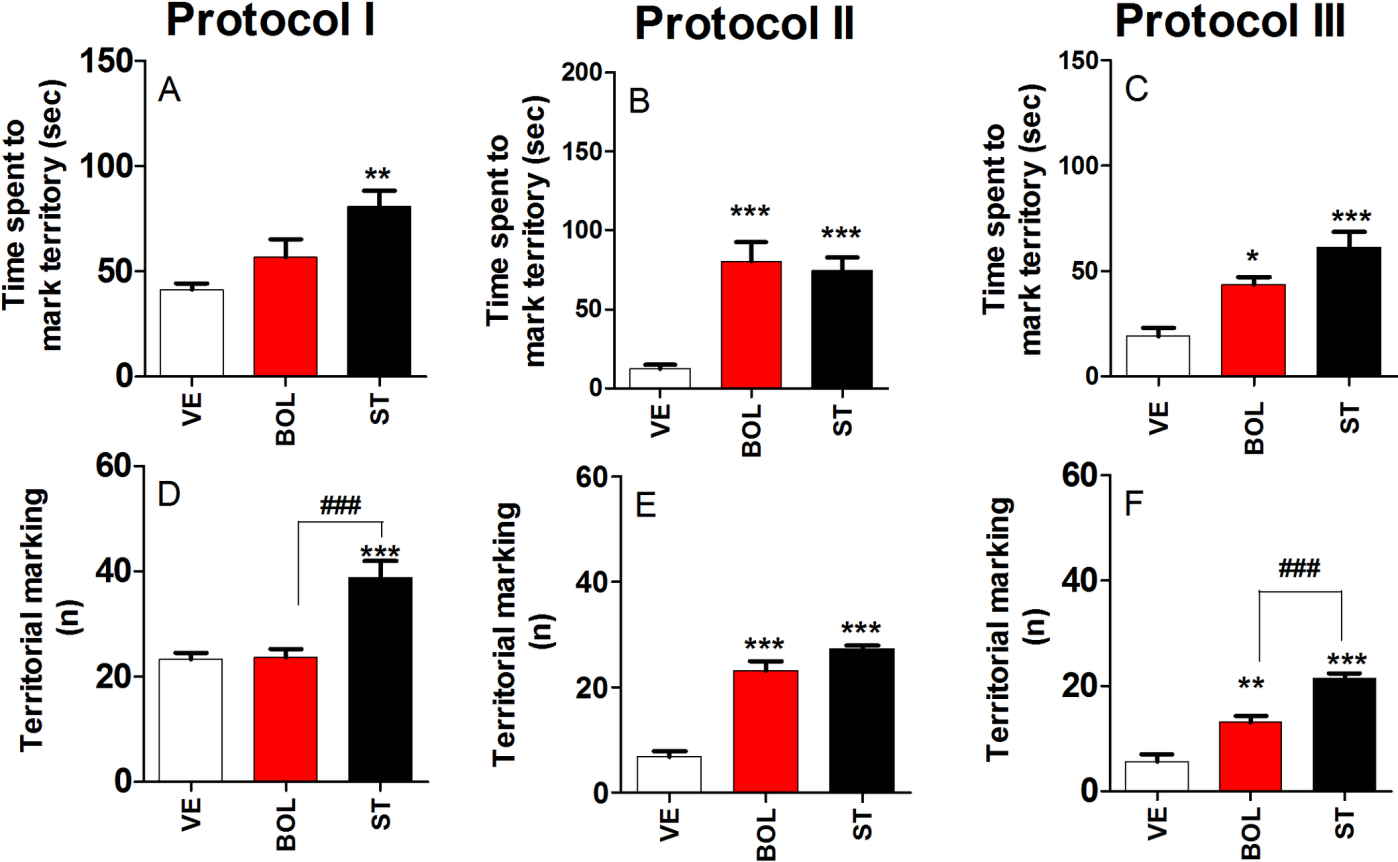
Dominant behavior over territory in rats treated intramuscularly with vehicle (olive oil, 0.2 ml), boldenone (BOL), or stanazolol (ST) once a week according to Protocol I (4 weeks, 5 mg/kg), Protocol II (8 weeks, 2.5 mg/kg), or Protocol III (12 weeks, 1.25 mg/kg). Time spent marking territory for Protocol I (A), II (B), and III (C) and territory-marking events for Protocol I (D), II (E), and III (F). *Denotes significant difference from the vehicle group. # Denotes significant difference between BOL and ST groups.

Fig 4 shows the time spent to mark territory (A, B, and C) and the number of these events (D, E, and F) after treatment with BOL or ST according to Protocol I, II, and III. Rats treated with ST according to Protocol I, II, and III spent an increased amount of time marking their territory [F_(2,29)_ = 8.815, p<0.01, graph A; F_(2,29)_ = 20.38, p<0.001, graph B; and F_(2,29)_ = 14.73, p<0.001, graph C, respectively], which was not observed for rats treated with BOL according to Protocol I. In addition, rats treated with BOL according to Protocol II and III spent significantly more time marking their territory [F_(2,29)_ = 20.38, p<0.001, graph B; and F_(2,29)_ = 14.73, p<0.01, graph C, respectively].

Rats treated with ST according to Protocol I, II, and III displayed a significant increase in the number of territorial-marking events [F_(2,29)_ = 16.37, p<0.001, graph D; F_(2,29)_ = 74.68, p<0.001, graph E; and F_(2,29)_ = 42.89, p<0.001, graph F, respectively], which was not observed for rats treated with BOL according to Protocol I [graph D]. However, these numbers were significantly increased in rats receiving BOL according to Protocol II and III [F_(2,29)_ = 74.68, p<0.001, graph E; and F_(2,29)_ = 42.89, p<0.001, graph F, respectively].

### Submissive behavior of rats treated with BOL and ST according to Protocol I, II, and III

**Fig 5:**
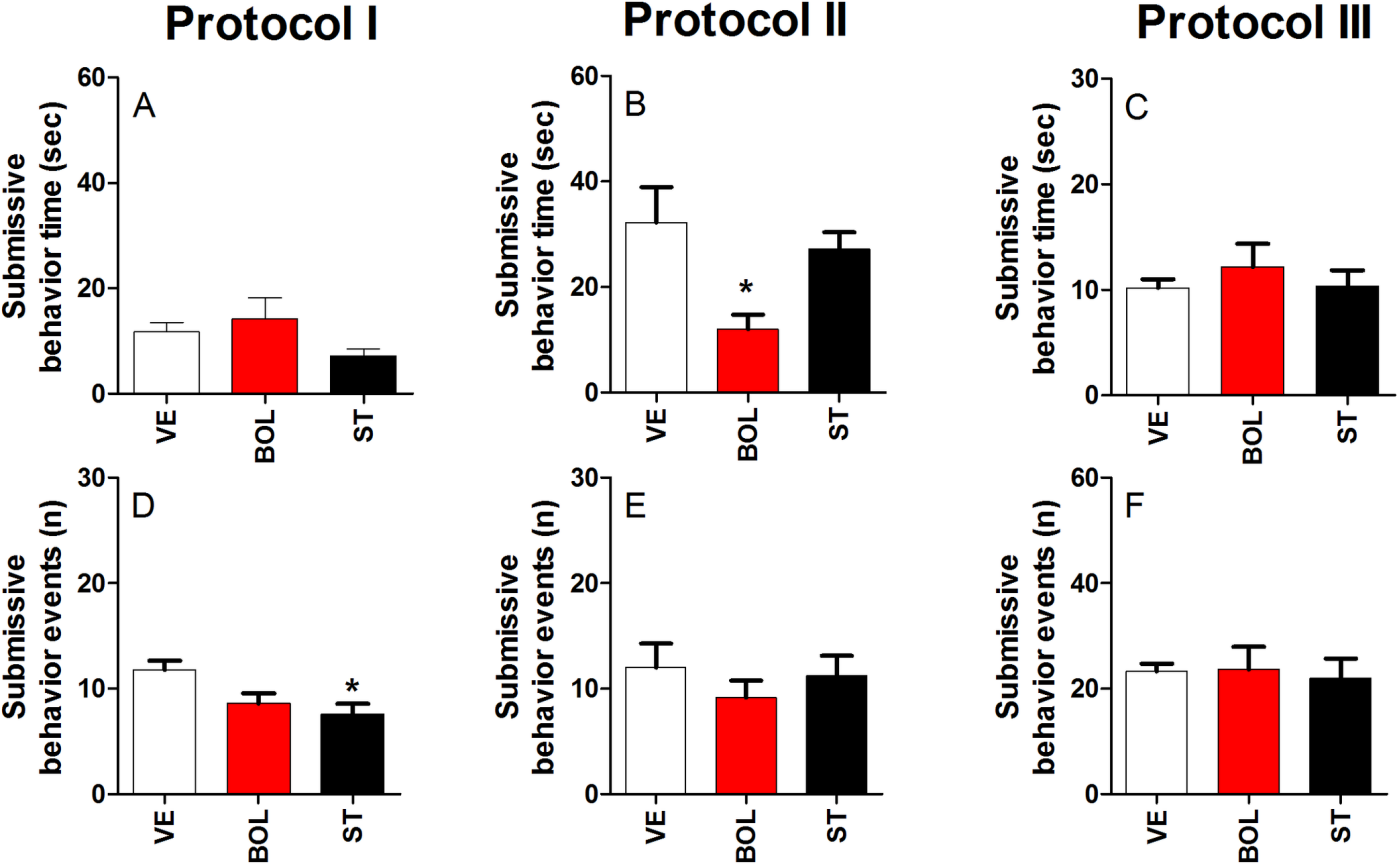
Submissive behavior of rats (n = 10) treated intramuscularly with vehicle (olive oil, 0.2 ml), boldenone (BOL), or stanazolol (ST) once a week according to Protocol I (4 weeks, 5 mg/kg), Protocol II (8 weeks, 2.5 mg/kg), or Protocol III (12 weeks, 1.25 mg/kg). Submissive behavior time for Protocol I (A), II (B), and III (C) and submissive behavior events for Protocol I (D), II (E), and III (F). *Denotes significant difference from the vehicle group. # Denotes significant difference between BOL and ST groups.

Fig 5 shows the time (A, B, and C) and events (D, E, and F) of submissive behavior after treatment with BOL or ST according to Protocol I, II, and III. Rats treated with ST according to Protocol I did not show a change in submissive behavior time [graphs A, B, and C]. Treatment with BOL decreased submissive behavior time only when administered according to Protocol II [F_(2,29)_ = 5.164, p<0.05, graph B], while treatment with ST reduced the number of submissive behavior events only when administered according to Protocol I [F_(2,29)_ = 5.045, p<0.05, graph D].

### Aggressive behavior of rats treated with BOL and ST according to Protocol I, II, and III

**Fig 6:**
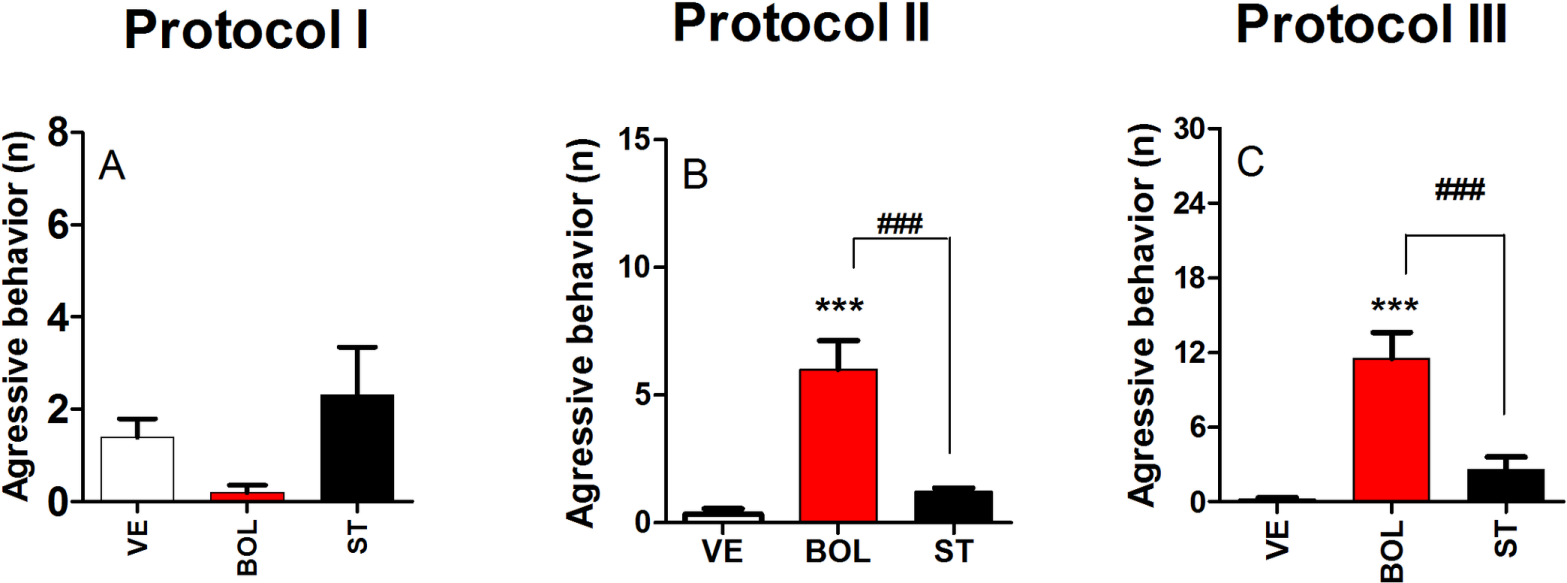
Aggressive behavior of rats (n = 10) treated intramuscularly with vehicle (olive oil, 0.2 ml), boldenone (BOL), or stanazolol (ST) once a week according to Protocol I (4 weeks, 5 mg/kg, graph A), Protocol II (8 weeks, 2.5 mg/kg, graph B), or Protocol III (12 weeks, 1.25 mg/kg, graph C). *Denotes significant difference from the vehicle group. # Denotes significant difference between BOL and ST groups.

Fig 6 shows aggressive behavior events after treatment with BOL or ST according to Protocol I, II, and III. Rats receiving ST according to Protocol I, II, and III did not show changes in aggressive behavior (graph A, graph B, and graph C, respectively). However, an increase in aggressive behavior was observed for rats treated with BOL according to Protocol II and III [F_(2,29)_ = 23.38, p<0.001, graph B; and F_(2,29)_ = 21.92, p<0.001, graph C, respectively].

### Object recognition task indices and acetylcholinesterase (AChE) activity in the brain of rats treated with BOL and ST according to Protocol I, II, and III

**Fig 7:**
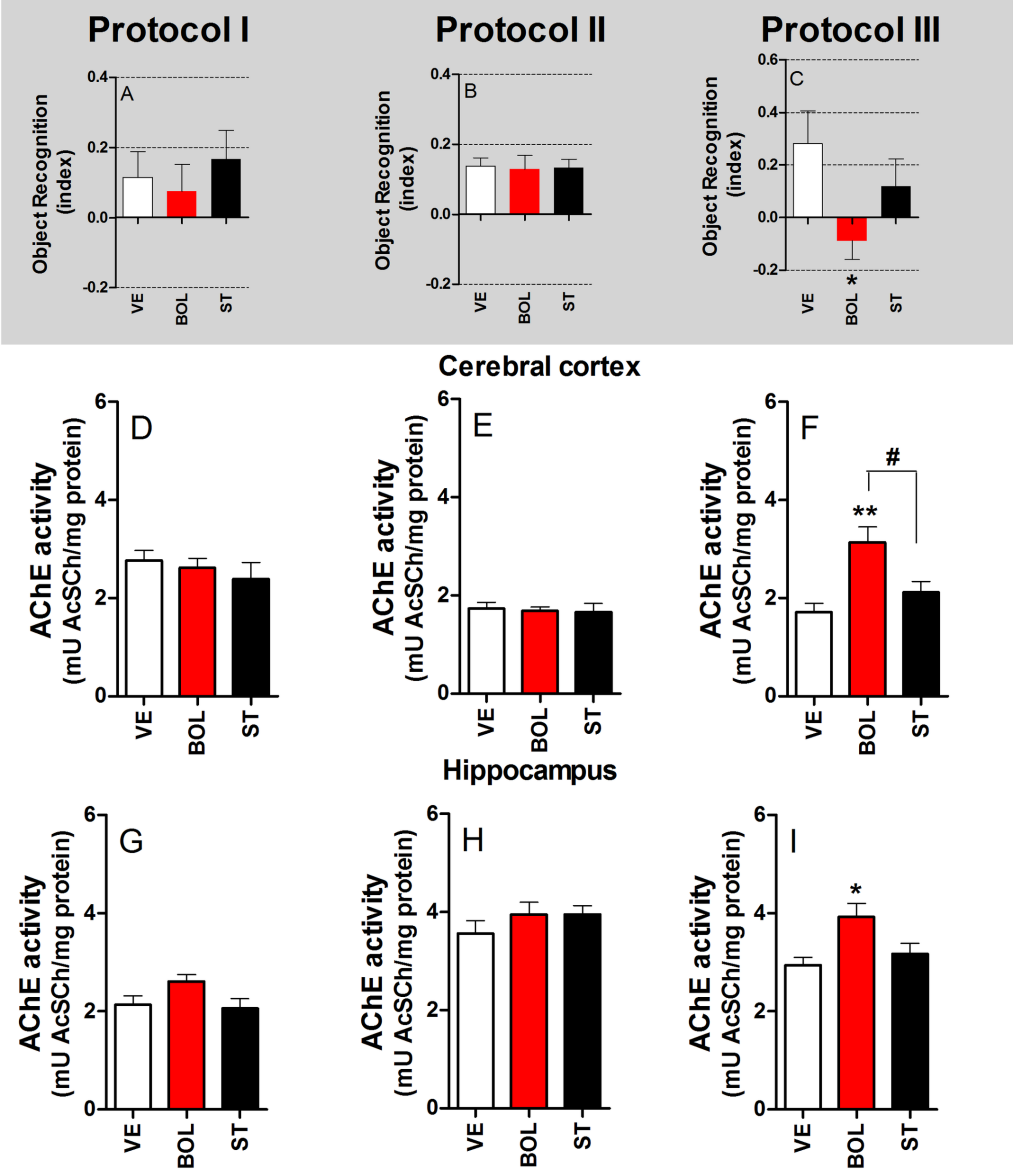
Object recognition task index and acetylcholinesterase (AChE) activity in the brain of rats (n = 10) treated intramuscularly with vehicle (olive oil, 0.2 ml), boldenone (BOL), or stanazolol (ST) once a week (intramuscular) according to Protocol I (4 weeks, 5 mg/kg, graph A), Protocol II (8 weeks, 2.5 mg/kg, graph B), or Protocol III (12 weeks, 1.25 mg/kg, graph C). Object recognition task index for Protocol I (A), II (B), and III (C). AChE activity in the cerebral cortex for Protocol I (D), II (E), and III (F) and hippocampus for Protocol I (G), II (H), and III (I). *Denotes significant difference from the vehicle group. # Denotes significant difference between BOL and ST groups.

Fig 7 shows the index value of the object recognition task of the rats, as well as the AChE activity in the CC and HC of rats treated with BOL and ST according to Protocol I, II, and III. Rats treated with BOL and ST according to Protocol I and II showed no significant differences in the object recognition task index or AChE activity in the CC and HC [graphs A and B, D and E, and G and H, respectively). However, rats treated with BOL according to Protocol III had reduced index scores in the object recognition task [F_(2,29)_ = 3.601, p<0.05, graph C] and increased AChE activity in the CC [F_(2,29)_ = 8.321, p<0.01, graph F] and hippocampus [F_(2,29)_ = 4.968, p<0.05, graph I].

### Oxidative stress parameters in the CC of rats treated with BOL or ST according to Protocol I, II, and III

**Fig 8:**
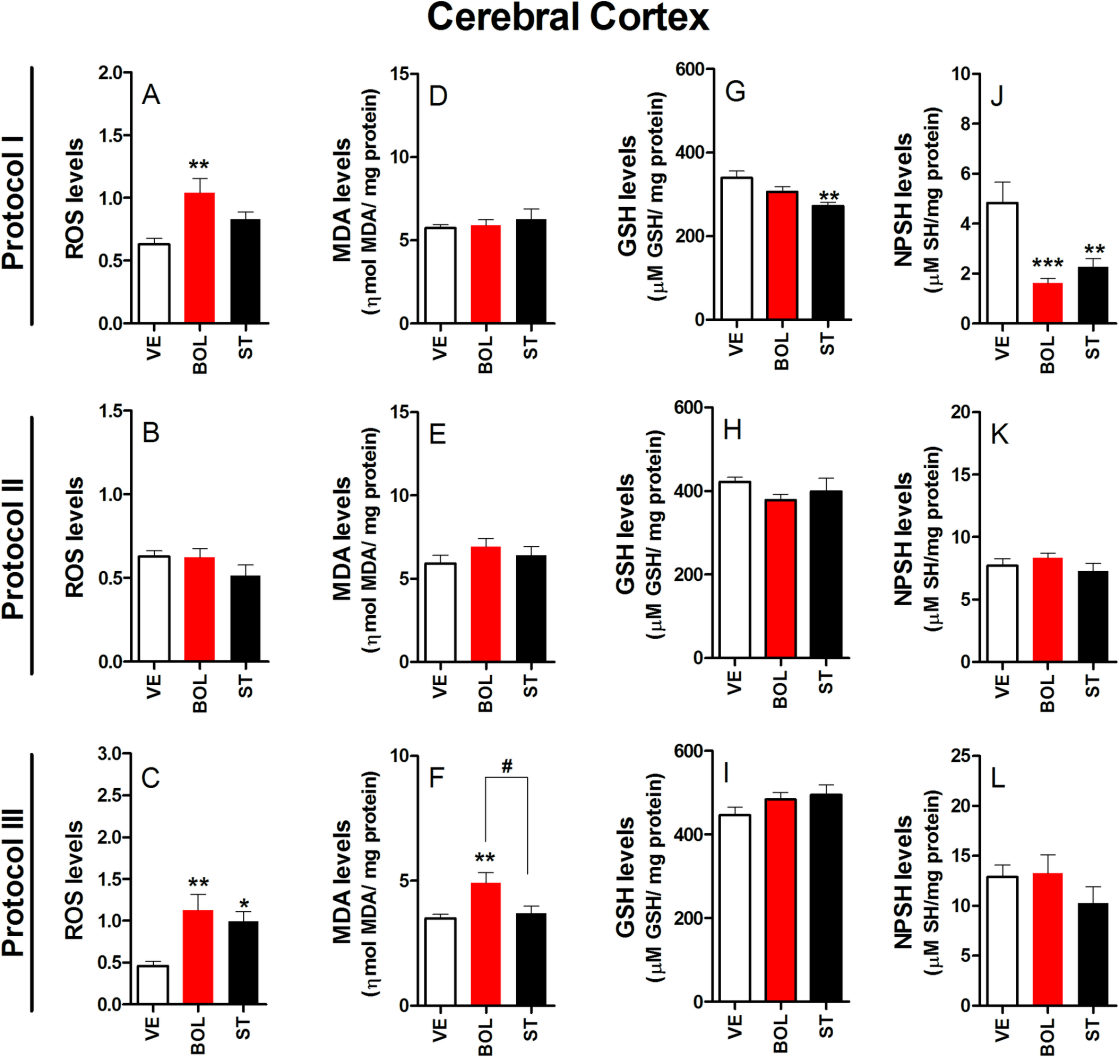
Parameters of oxidative stress in the cerebral cortex of rats (n = 10) treated intramuscularly with vehicle (olive oil, 0.2 ml), boldenone (BOL), or stanazolol (ST) once a week according to Protocol I (4 weeks, 5 mg/kg, graph A), Protocol II (8 weeks, 2.5 mg/kg, graph B), or Protocol III (12 weeks, 1.25 mg/kg, graph C). Reactive oxygen species (ROS) production for Protocol I (A), II (B), and II (C); malondialdehyde (MDA) levels for Protocol I (D), II (E), and III (F); reduced glutathione (GSH) levels for Protocol I (G), II (H), and III (I); non-protein thiol (NPSH) levels for Protocol I (J), II (K), and III (L). *Denotes significant difference from the vehicle group. # Denotes significant difference between BOL and ST groups.

Increased levels of reactive oxygen species (ROS) were observed in rats treated with BOL according to Protocol I [F_(2,29)_ = 7.547, p<0.01, graph A] and III [F_(2,29)_ = 7.910, p<0.01, graph C] (Fig 8). In contrast, treatment with ST resulted in increased ROS levels only when administered according to Protocol III [F_(2,29)_ = 7.910, p<0.05, graph C]. No significant differences in ROS levels were observed in rats receiving BOL and ST according to Protocol II [F_(2,29)_ = 1.550, graph B]. A significant increase in malondialdehyde (MDA) levels was observed in rats treated with BOL according to Protocol III [F_(2,29)_ = 6.561, p<0.01, graph F]; however, no significant differences in MDA levels were observed in rats treated with BOL and ST according to Protocol I [F_(2,29)_ = 0.490, graph D] and II [F_(2,29)_ = 1.106, graph E]. Reduced glutathione (GSH) levels were only observed in rats treated with ST according to Protocol I [F_(2,29)_ = 6.495, p<0.01, graph G]. No significant differences were observed in the GSH levels of rats receiving BOL and ST according to Protocol II [F_(2,29)_ = 0.945, graph H] and III [F_(2,29)_ = 1.537, graph I]. Non-protein thiols (NPSH) levels decreased in rats receiving BOL and ST according to Protocol I [F_(2,29)_ = 10.09, p<0.001, graph J]. No significant differences were observed in rats receiving these two drugs according to Protocol II [F_(2,29)_ = 0.968, graph K] and III [F_(2,29)_ = 1.126, graph L].

### Oxidative stress parameters in the HC of rats treated with BOL or ST according to Protocol I, II, and III

**Fig 9:**
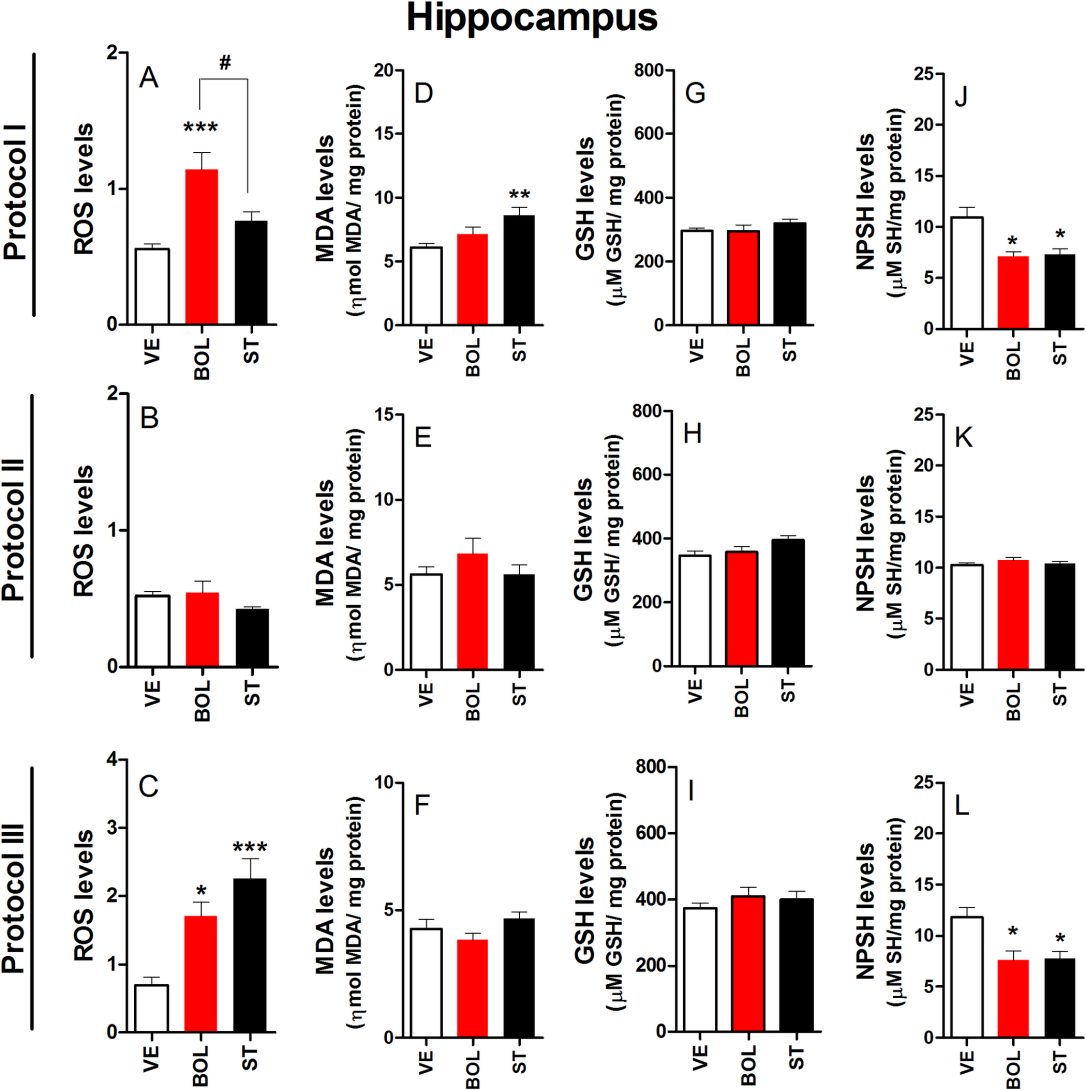
Parameters of oxidative stress in the hippocampus of rats (n = 10) treated intramuscularly with vehicle (olive oil, 0.2 ml), boldenone (BOL), or stanazolol (ST) once a week according to Protocol I (4 weeks, 5 mg/kg, graph A), Protocol II (8 weeks, 2.5 mg/kg, graph B), or Protocol III (12 weeks, 1.25 mg/kg, graph C). Reactive oxygen species (ROS) production for Protocol I (A), II (B), and II (C); malondialdehyde (MDA) levels for Protocol I (D), II (E), and III (F); reduced glutathione (GSH) levels for Protocol I (G), II (H), and III (I); non-protein thiol (NPSH) levels for Protocol I (J), II (K), and III (L). *Denotes significant difference from the vehicle group. # Denotes significant difference between BOL and ST groups.

Fig 9 shows that ROS levels increased in rats treated with BOL according to Protocol I [F_(2,29)_ = 12.21, p<0.001, graph A] and III [F_(2,29)_ = 12.67, p<0.05, graph C]. In contrast, ROS levels increased in rats treated with ST according to Protocol III only [F_(2,29)_ = 12.67, p<0.001, graph C]. No significant differences in ROS levels were observed in rats treated with ST according to Protocol II [F_(2,29)_ = 0.176, graph B]. Increased levels of MDA were observed in rats treated with ST according to Protocol I [F_(2,29)_ = 6.610, p<0.01, graph D]. There were no significant differences in MDA levels in rats treated with BOL and ST according to Protocol II [F_(2,29)_ = 1.113, graph E] and III [F_(2,29)_ = 1.1973, graph F]. GSH levels were unchanged in rats treated with BOL and ST according to Protocol I [F_(2,29)_ = 0.863, graph G], II [F_(2,29)_ = 2.347,graph H], and III [F_(2,29)_ = 0.675, graph I]. NPSH levels decreased in rats treated with BOL and ST according to Protocol I [F_(2,29)_ = 7.682, p<0.01, graph J] and III [F_(2,29)_ = 6.902, p<0.01, graph L]. In contrast, no significant differences in these levels were observed in rats treated with BOL and ST according to Protocol II [F_(2,29)_ = 1.202, graph K].

## Discussion

In the present study, we measured the physiological effects of BOL and ST using three different treatment protocols. Protocol I consisted of a dose higher than the recommended dose administered over a shorter time interval. Protocol II consisted of a moderate dose administered over an intermediate period, and Protocol III consisted of a reduced dose administered over an extended period [26]. These protocols were based on steroid user groups known to use widely ranging steroid doses and treatment durations [1, 27].

Our research focused on comparing the behavioral changes in rats receiving BOL and ST according to the three protocols described. Locomotion parameters were unchanged in the rats receiving BOL and ST, regardless of the treatment protocol used. In contrast, Bronson and Bronson [28] and colleagues [29] have reported that female mice treated with a combination of AAS at low and high doses for either 9 weeks or 6 months (high dose only) exhibited significantly reduced spontaneous activity in a running wheel relative to that observed for the controls. Further, these authors hypothesized that female-specific effects of AAS on locomotion might reflect AAS antagonism of estrogen-induced spontaneous activity [28, 29].

Here, we showed that the anxiety levels were significantly increased in the rats receiving BOL and ST according to Protocol I since these rats spent less time in the open arms of the elevated plus maze. Bitran and colleagues performed the first study of the effects of AAS on anxiety [30]. Another study showed that high doses of testosterone propionate induced an anxiogenic effect after 6 days of treatment; however, after 14 days of treatment the animals did not behave differently from those in the control group [31]. These results are indicative of a transitional character treatment, which might explain the fact that the relatively longer treatments at a relatively low dose used in our study did not increase anxious behavior. Nevertheless, Minkin and colleagues showed that nandrolone administered for 8 weeks increased anxious behavior, contradicting the findings of anxiolytic activity of some steroids [32].

Dominant behavior over the intruder and territorial dominance increased in all protocols tested, while submissive behavior was not affected by the BOL and ST treatments according to Protocol I and III. However, submissive behavior events decreased only when the animals were exposed to the higher dose of ST (PI). Further, the treatment with BOL according to Protocol II and III affected aggressiveness. The findings of Kalinine et al. show that a neural circuit composed of several regions including the prefrontal cortex, amygdala, HC, hypothalamus, anterior cingulated cortex, and other interconnected structures is implicated in the regulation of emotions [33]. Furthermore, it is plausible that functional or structural abnormalities in these regions can increase the susceptibility to impulsive aggression and violence [34]. AAS also affect aggressive behavior in rodents [35]. Single compounds such as testosterone and testosterone propionate increase dominance and aggression in healthy adult male rats and mice [36, 37]. Further, adolescent male Syrian hamsters exposed to a cocktail of AAS for 2 or 4 weeks showed increased aggressive behavior [10]. Consequently, the choice of a specific protocol for drug administration (both time and dose dependent), as well as the type of AAS used can affect the brain differently, resulting in a greater or lesser degree of dominance and aggressiveness.

The array of physiological and behavioral effects of these chemically disparate drugs is vastly compounded not only by complexity in the patterns of self-administration, but also by the heterogeneity of the subjects that take these drugs [38, 39]. Symptoms resulting from the chronic use of supra-therapeutic doses of AAS include mania, increased anxiety, irritability, extreme mood swings, and abnormal levels of aggression, depression, and even suicide. In particular, individuals self-administering the highest doses of AAS have elevated scores on the “Symptom Check List-90”, a self-report system that includes a number of different dimensions of anxiety [40]. These findings support our data that clearly show different behavioral responses of the rats treated with BOL and ST depending on the exposure duration and the dose. However, to investigate the effect of BOL and ST on cognitive processes, additional experimental techniques can be used. Since gonadal hormones are known to play a crucial role in cognitive processes such as spatial learning performance [41] and extinction responses in a passive avoidance task [42], it is conceivable that BOL and ST also influence cognitive functions. In particular, the current literature contains several controversial data regarding the absence of cognitive disorders in animals treated with AAS [43, 44], while other studies have clearly shown that individuals abusing AAS have a high risk of developing cognitive disorders and important morphological changes in the amygdala, HC, and forebrain [45, 46]. Thus, the present study evaluated memory performance of rats treated with BOL and ST in the object recognition task. Only rats receiving BOL according to Protocol III had a significant memory deficit compared to that of rats in the control and ST groups. Therefore, the use of BOL at a reduced dose but for a relatively long duration had a higher impact on learning and memory compared to that observed for ST treatment.

The biochemical mechanisms underlying the effects of AAS are still poorly understood. The mesocorticolimbic dopaminergic pathway is considered to play an important role in the reinforcement circuitry of the brain [47], and a connection between AAS and central dopaminergic and serotoninergic activity has been found in animal studies [48, 49]. However, only little information is available to explain the potential role of the cholinergic system in the biochemical mechanisms underlying the CNS effects of AAS. Such information is, however, especially important given that the cholinergic system plays an essential role in memory formation and learning [50, 51], and perhaps also in the behavioral changes we observed according to the type, treatment duration, and dose of the AAS used. Because acetylcholine coordinates the neuronal network response, modulation of the cholinergic system is an essential mechanism underlying complex behaviors; stimulation of presynaptic nicotinic acetylcholine receptors can increase the release of glutamate, ˠ-aminobutyric acid (GABA), dopamine, acetylcholine, norepinephrine, and serotonin [52, 53]. AChE is an important enzyme that regulates the concentration of acetylcholine in the synaptic cleft. We observed an increase in AChE activity in the CC and HC of animals treated with BOL (protocol III). These results are supported by those of other studies that have shown that the anabolic androgenic steroid methandrostenolone changes the expression of neuronal growth factor (NGF) and its receptors while reducing the activity of choline acetyltransferase (ChAT). Methandrostenolone also induces the synthesis of acetylcholine in the basal forebrain and impairs behavioral performance in the Morris water maze task [46].

Experimental studies in animals suggest that the redox status may be involved in AAS-induced neurotoxicity [54]. To the best of our knowledge, our results are the first to demonstrate that both BOL and ST change ROS, MDA, GSH, and NPSH levels in the CC and HC of rats. However, we found no significant changes in the CC and HC of rats that had received BOL and ST according to Protocol II. We hypothesize that both BOL and ST cause a physiological adaptation via changing of the redox status homeostasis.

The main limitations of this preliminary study were that we did not explore the underlying molecular mechanisms of the effects observed, and that we did not assess the expression of superoxide dismutase and glutathione peroxidase and reductase in the brain. Measuring antioxidant enzymes in brain tissue may aid in finding a potential correlation between the ROS, GSH, and NPSH levels in the treatment protocols we used. However, we found that a high dose or chronic use of BOL or ST changed oxidative stress parameters, resulting in deleterious effects on the HC and CC. Similar results were reported by Tugyan and colleagues who demonstrated that nandrolone increased MDA levels and reduced GPX activity, thereby negatively affecting the consequences of brain injury [55]. Holmes and colleagues suggested that androgens are neuroprotective at minimal oxidative stress levels, but that they exacerbate oxidative stress damage at elevated oxidative stress levels [56]. Additionally, Cunningham et al. demonstrated that in a preexisting oxidative stress environment, androgens could further exacerbate oxidative stress damage [57, 58].

In summary, our findings highlight that the impact of BOL and ST on cognition, anxiety, and social interaction depends on their dose and exposure duration. Furthermore, it is plausible that there is a correlation between the use of anabolic steroids and increased oxidative stress in the brain. More studies are necessary to understand the mechanisms by which BOL and ST affect brain function.
